# Supplementary figures and images for: SARS-CoV-2 hijacks neutralizing dimeric IgA for nasal infection and injury in Syrian hamsters1
Source: Emerg Microbes Infect. 2023 Aug 21;12(2):2245921. doi: 10.1080/22221751.2023.2245921 (PMC10444022; doi:10.1080/22221751.2023.2245921)

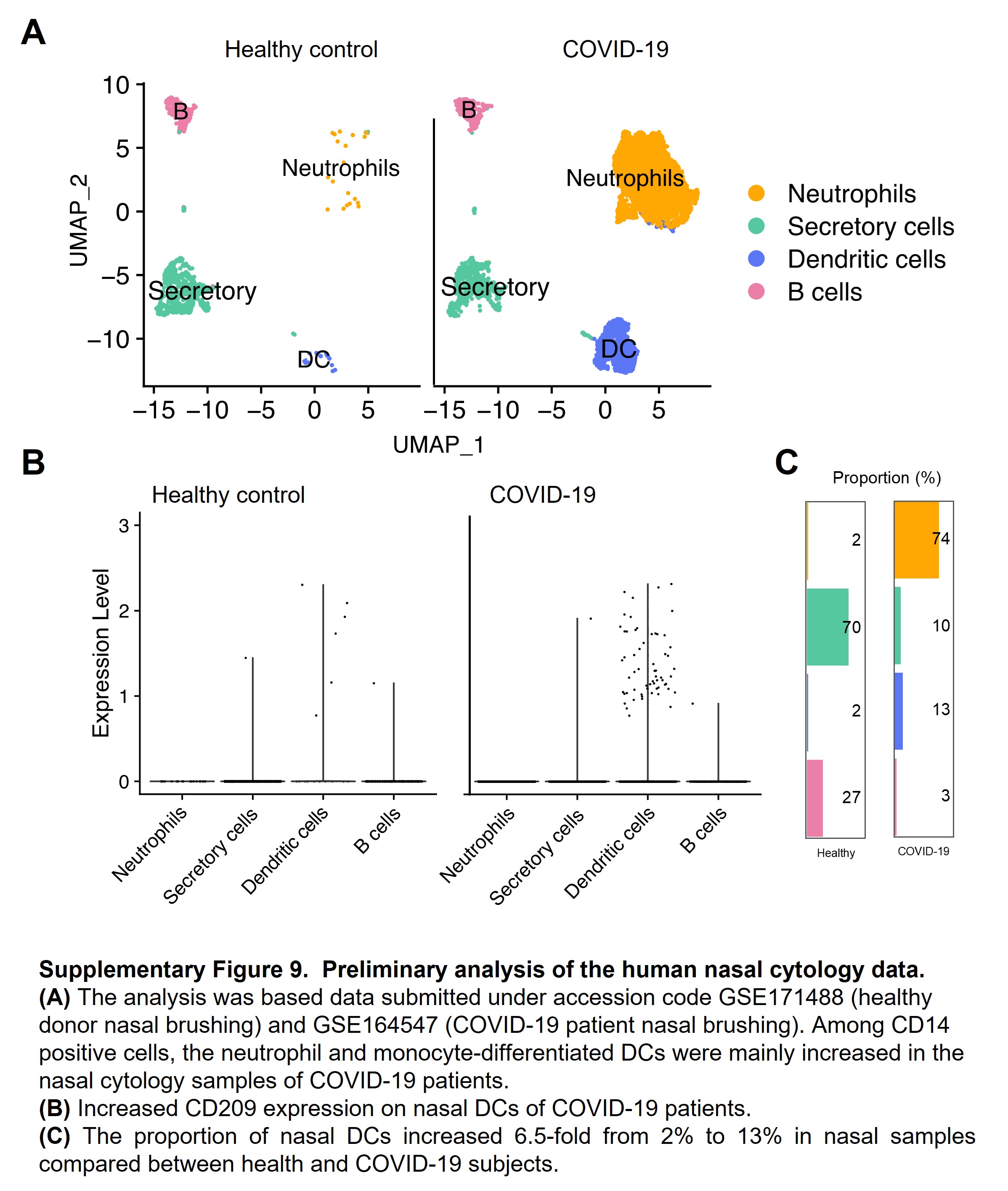

Supplement: Supplemental Material [file TEMI_A_2245921_SM3381.jpg]

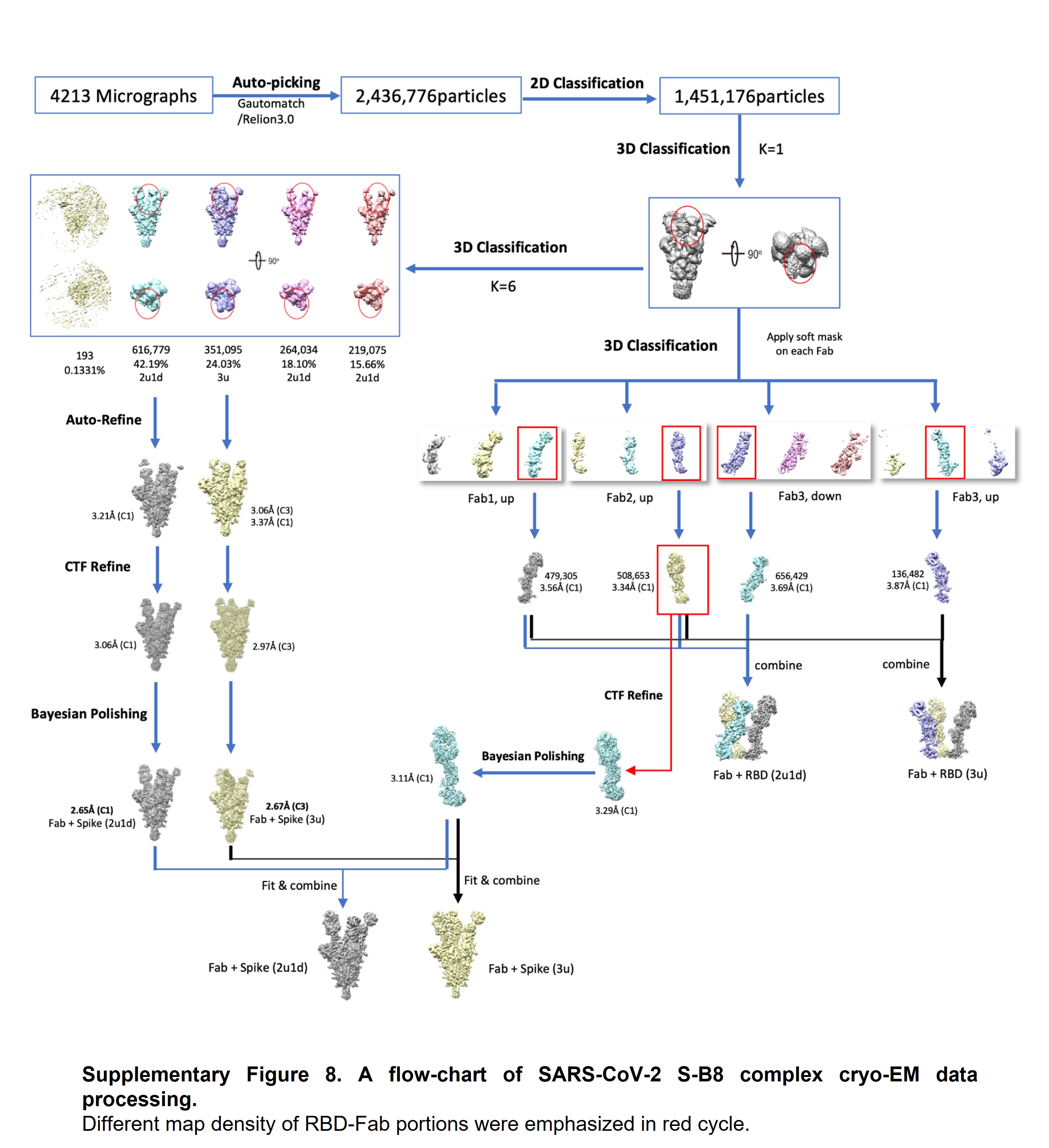

Supplement: Supplemental Material [file TEMI_A_2245921_SM3380.jpg]

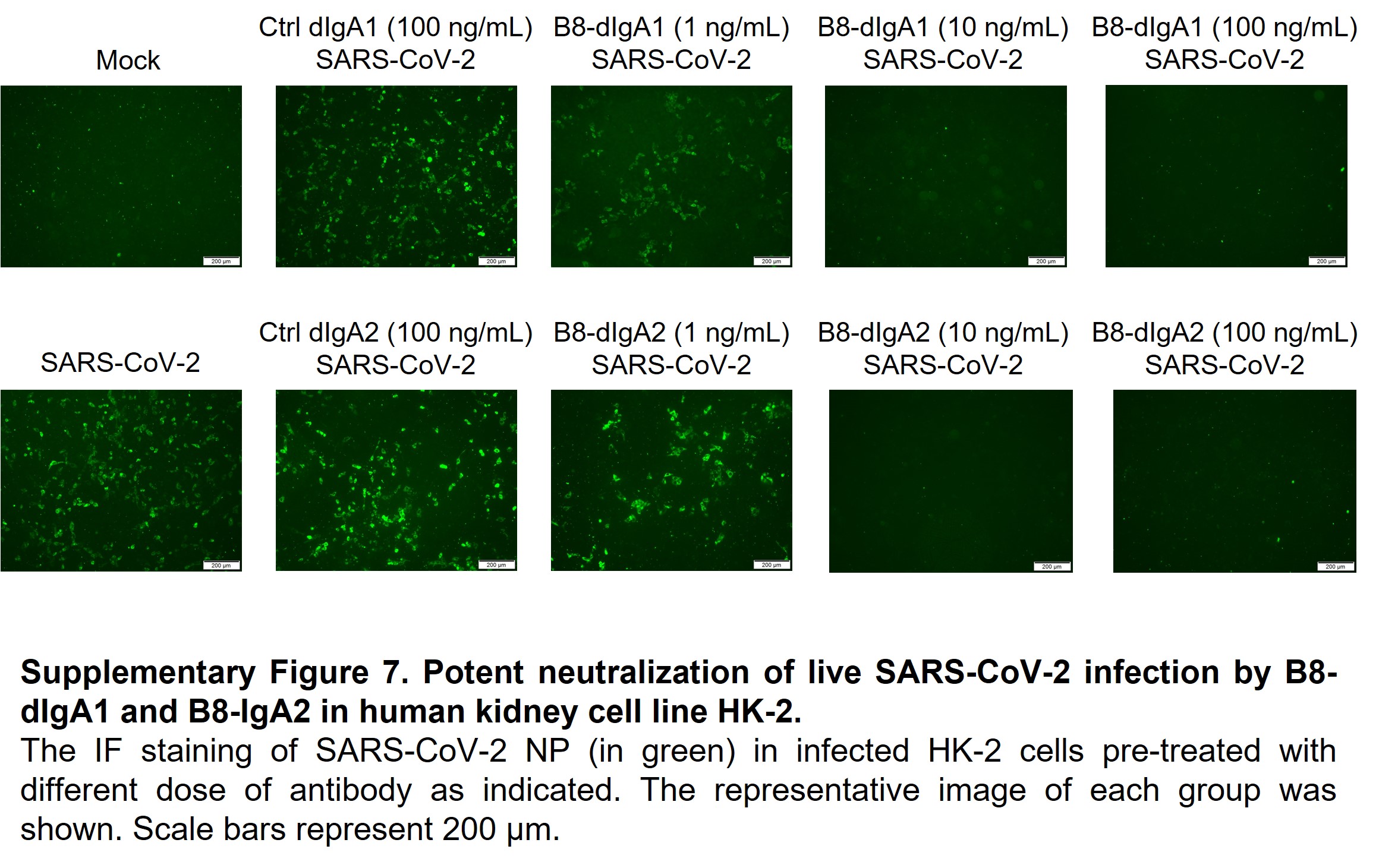

Supplement: Supplemental Material [file TEMI_A_2245921_SM3379.jpg]

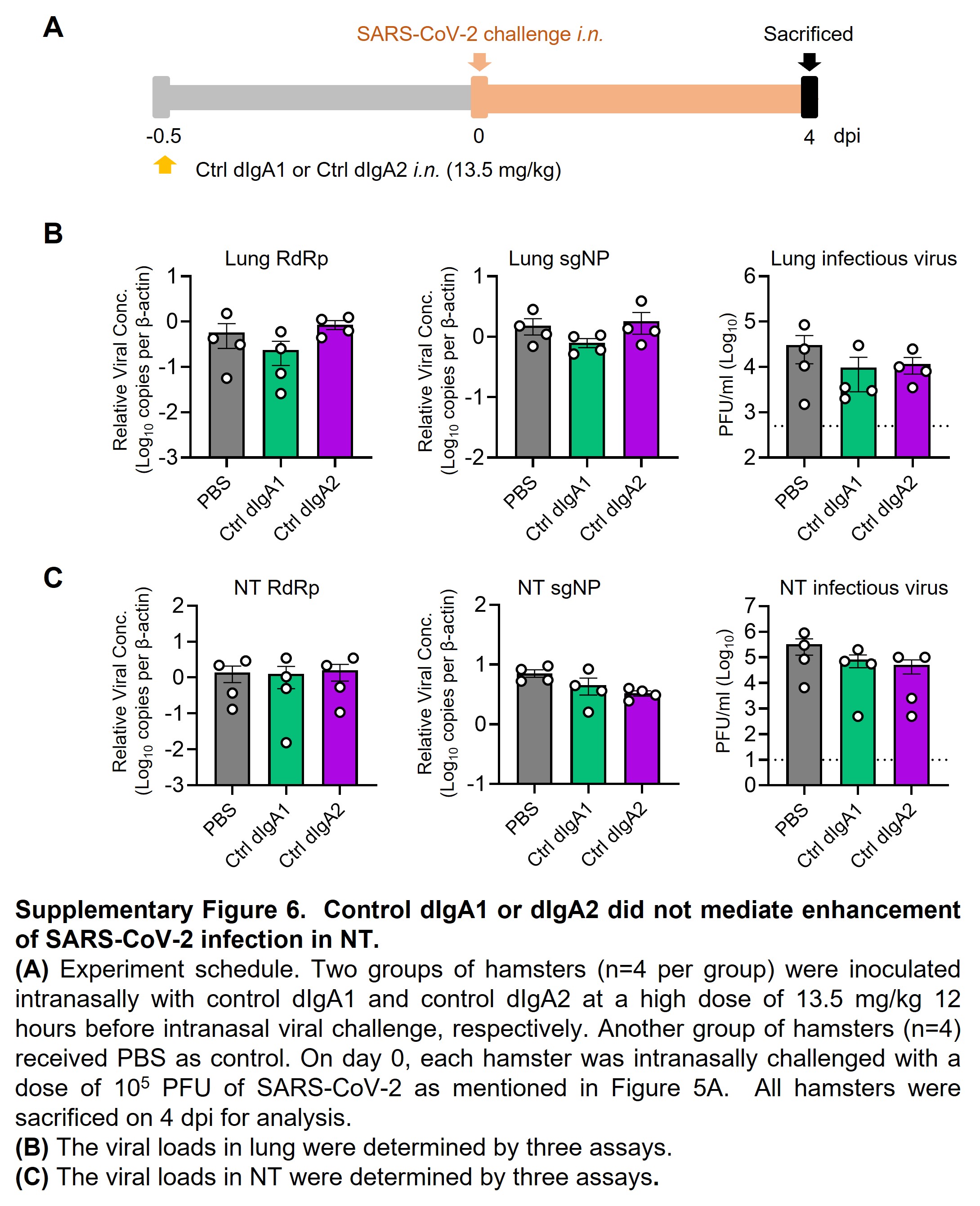

Supplement: Supplemental Material [file TEMI_A_2245921_SM3378.jpg]

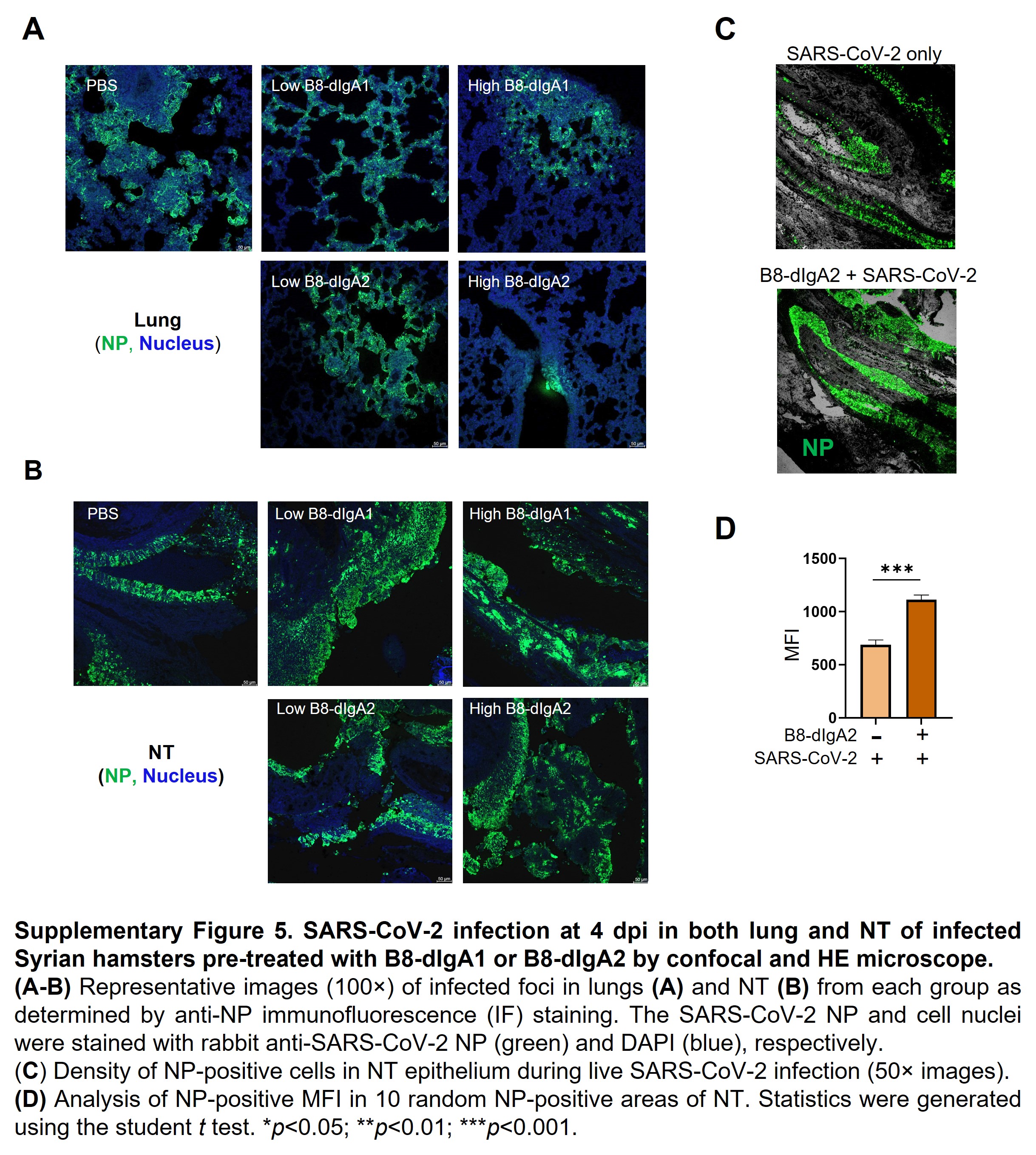

Supplement: Supplemental Material [file TEMI_A_2245921_SM3374.jpg]

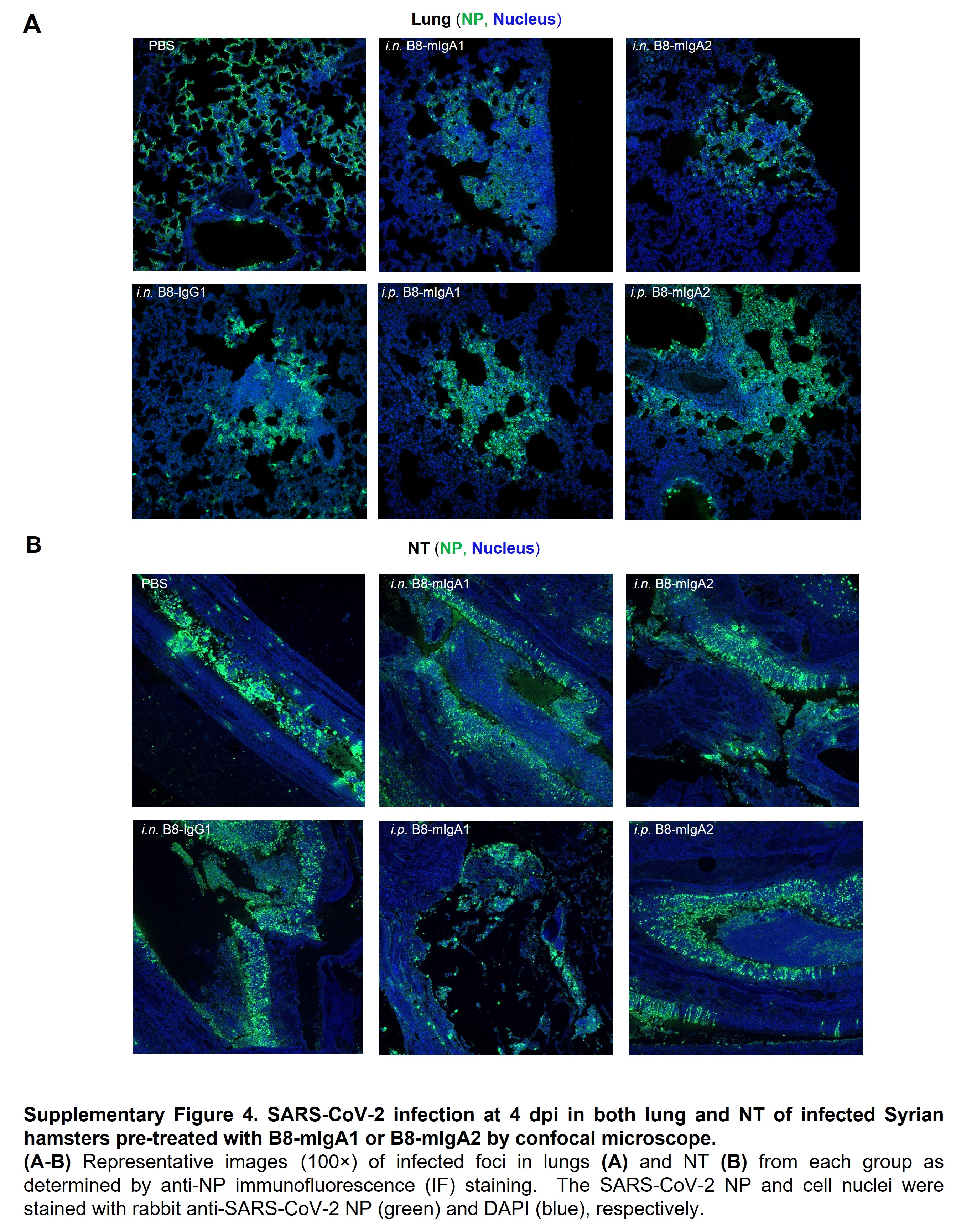

Supplement: Supplemental Material [file TEMI_A_2245921_SM3373.jpg]

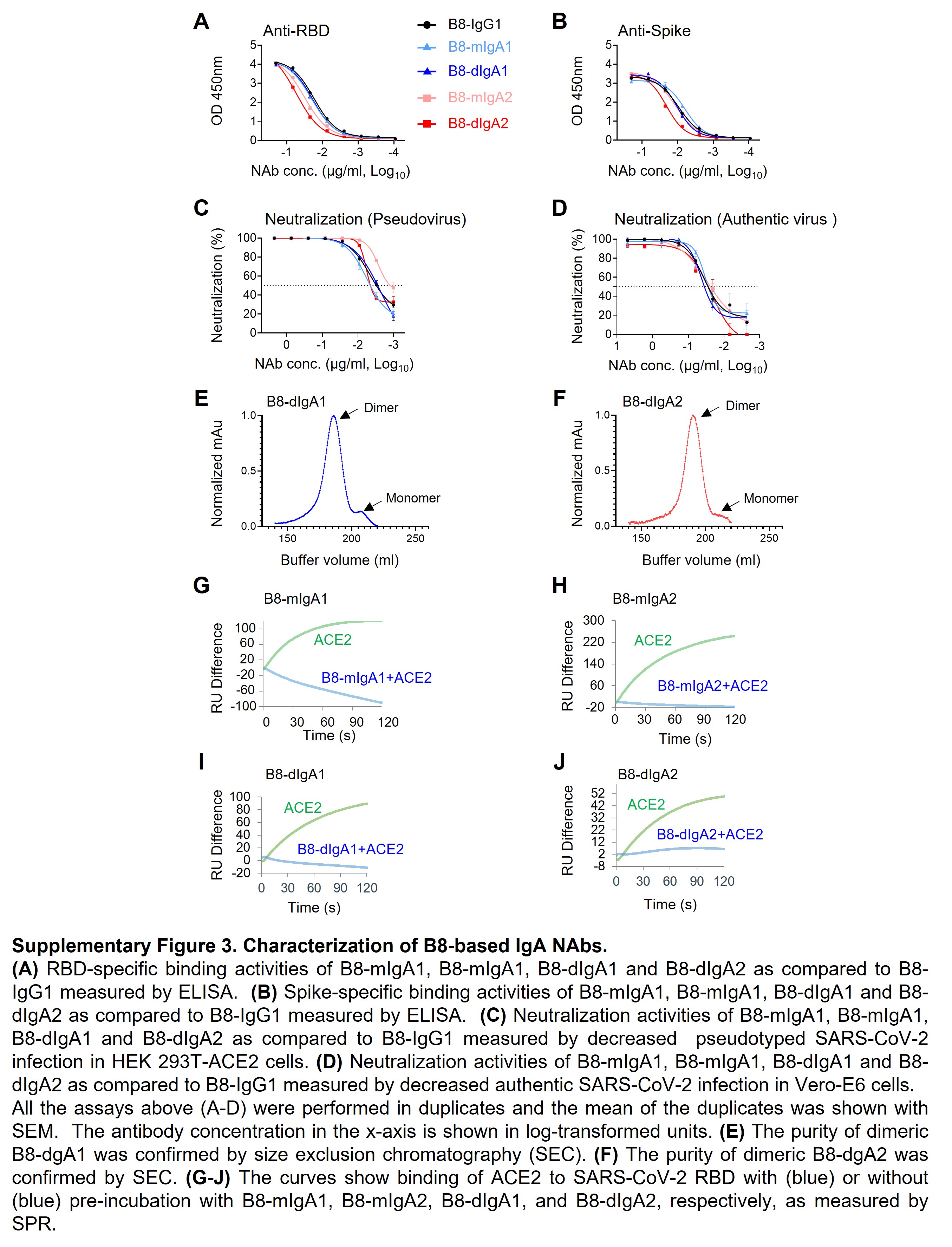

Supplement: Supplemental Material [file TEMI_A_2245921_SM3370.jpg]

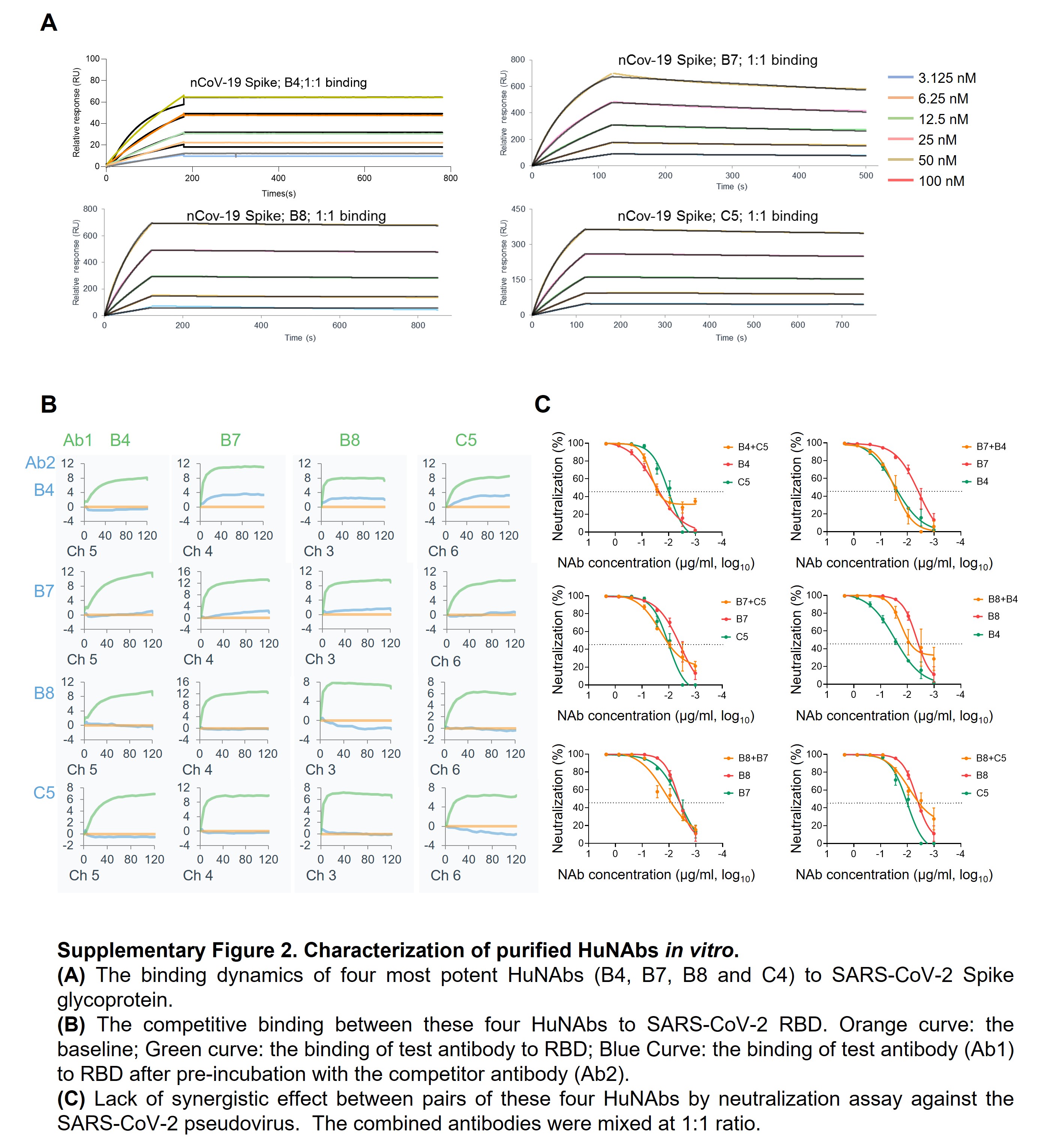

Supplement: Supplemental Material [file TEMI_A_2245921_SM3367.jpg]

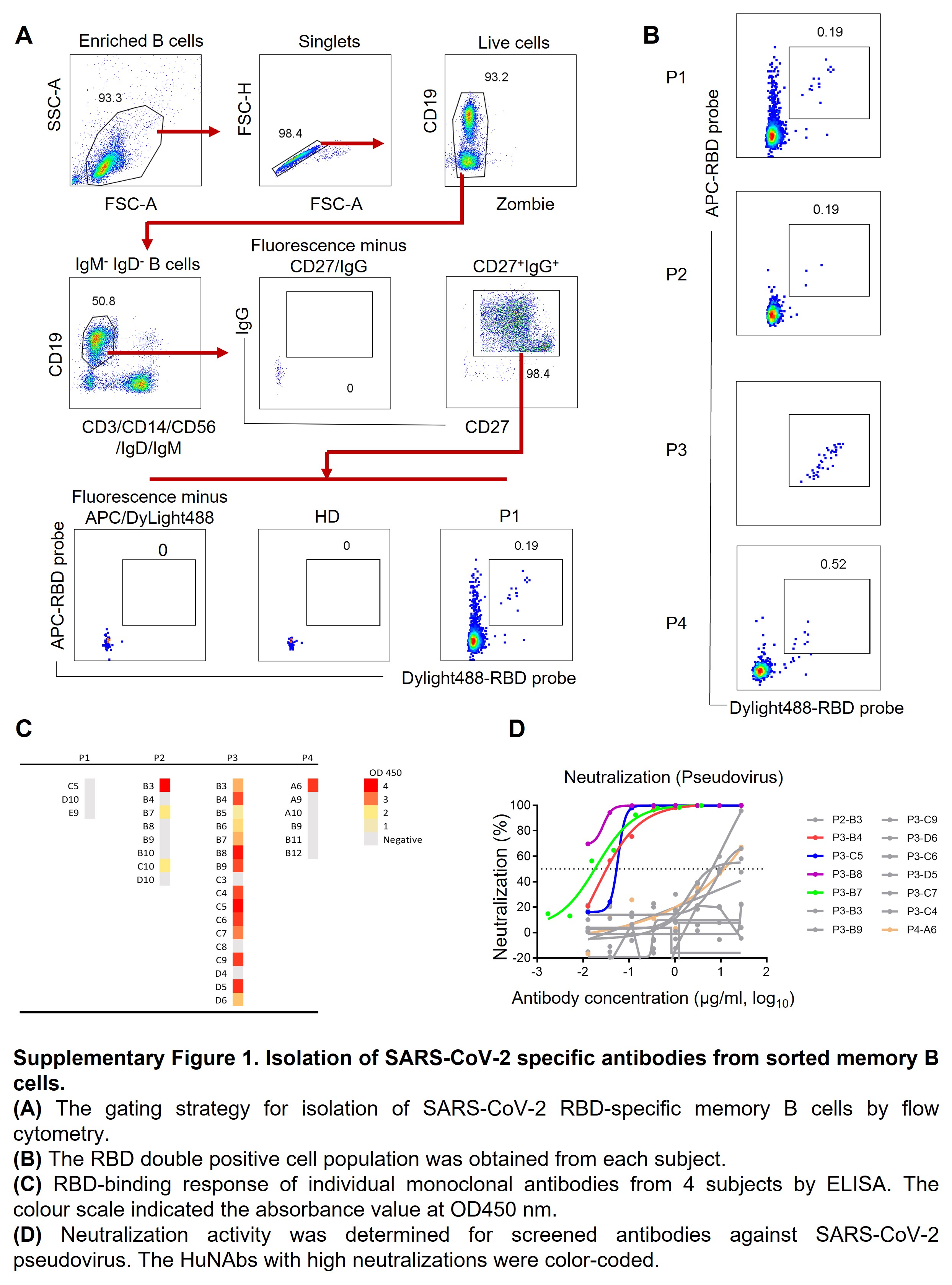

Supplement: Supplemental Material [file TEMI_A_2245921_SM3366.jpg]
